# Bioceramic dressing and its influence on intratubular penetration and bond strength of endodontic sealers

**DOI:** 10.4317/jced.62609

**Published:** 2025-06-01

**Authors:** Lívia Pacheco Rodrigues, Jefferson Ricardo Pereira, Fabiana Vieira Vier-Pelisser, Leonardo Thomasi Jahnke, Gabriel Barcelos Só, Ricardo Abreu da Rosa, Marcus Vinicius Reis Só

**Affiliations:** 1 School of Dentistry, Department of Conservative Dentistry, Federal University of Rio Grande do Sul – UFRGS, Porto Alegre, Brazil; 2 Department of Prosthodontics, Unisul - Universidade do Sul de Santa Catarina, Tubarão, SC, Brazil; 3 PhD in Endodontics from the Pontifical Catholic University of Rio Grande do Sul - PUCRS, Porto Alegra, Brazil

## Abstract

**Background:**

This study aimed to evaluate the influence of bioceramic root canal dressing (BRCD) BIO-C TEMP (BT) on intratubular penetrability through confocal laser scanning microscopy (CLSM) and bond strength (push out) of epoxy resin AH Plus (AH) and bioceramic Bio-C (BC) sealers and evaluate the dentin surface after removing the intracanal medication by scanning electron microscopy (SEM).

**Material and Methods:**

After biomechanical preparation, 48 bovine incisors were filled and evaluated for bond strength and intratubular penetrability. The groups were established by the type of sealer used: AH (n=24) or BC (n=24) and no use (0BT) (n=8) or use of root canal dressing for two different experimental times, 7 (7BT) (n=8) and 30 (30BT) (n=8) days. For SEM evaluation, five unfilled teeth were used: without BT (n=1), BT for 7 (n=2) and 30 (n=2) days. Kruskal-Wallis and T-tests were performed for bond strength analysis. Descriptive analysis was used in CLSM and SEM evaluations.

**Results:**

BT decreased the bond strength of AH and BC sealers in both experimental times (7 and 30 days) (*P*< 0.05). The intratubular penetration with AP was higher than that with BC. SEM showed remnants of BT on the dentin surface in both groups (7 and 30 days). The AH Plus sealer showed more evident intratubular penetration than the Bio-C sealer.

**Conclusions:**

It is not possible to remove a BRCD completely from the root canal. A bioceramic root canal dressing Bio-C Temp decreased the bond strength of both tested sealers, epoxy resin AH Plus and bioceramic Bio-C Sealer.

** Key words:**Root canal dressing, bioceramic, scanning electron microscopy, laser scanning confocal microscopy, sealer.

## Introduction

The presence of bacteria in areas inaccessible to instrumentation is the greatest challenge to the success of endodontic treatment (1). Therefore, aiming at bacterial reduction at levels compatible with tissue repair, using root canal dressing between visits helps disinfect the root canal system, increasing the predictability of treatment (2). The most commonly used medication for this purpose is calcium hydroxide (Ca(OH)2) due to its antimicrobial and repairing properties (3). However, it is known that this intracanal medication is not entirely removed from the interior of the root canal (4), which may interfere with the desired sealing of the filling material. This material can hinder the penetration and bonding of the filling sealers to the dentin, leading to failures in the filling and micro infiltration (5-10).

Recently, a bioceramic root canal dressing (BRCD), BIO-C TEMP (Angelus Industry Dental Products Ltda, Londrina, PR, Brazil), was launched. According to the manufacturer, this material comes in the form of a ready-to-use paste and has high alkalinity (pH: 12 ±1). It comprises calcium silicate, calcium aluminate, calcium oxide, calcium tungstate, titanium oxide and salicylate glycol ester.

The physicochemical properties of BIO-C TEMP were compared with calcium hydroxide (11). The new BRCD presented an alkaline pH, high calcium release, and good radiopacity but has a cytotoxic effect dependent on dose and exposure time and lacks tubular penetration. Alkalinity promotes a change in pH and dentin properties (12-14), which can lead to changes in the penetration and bonding of endodontic sealers of different compositions (15,16).

Another essential characteristic of bioceramic materials is their bioactivity: the ability to form apatite crystals. The calcium (Ca) and hydroxyl (OH) ions released from the material interact with the phosphate (P) present on site (11,17,18). Bioactive materials induce intratubular mineralization through the deposition of interfibrillar apatite, which functions as a mineralized barrier, reducing infiltration between the dentin material interface. Furthermore, they induce the formation of apatite crystals on the dentin surface (19-21).

Thus, the objective of this study was to evaluate the influence of BIO-C TEMP on the intratubular penetrability and bond strength of epoxy resin and bioceramic sealers and to evaluate the dentin surface after the medication was removed.

The null hypothesis of this study was that there would be no differences in the penetration and bond strength of bioceramic sealer with and without BIO-C TEMP.

## Material and Methods

- Ethical Considerations

This research was approved by the Research Committee of the School of Dentistry of the Federal University of Rio Grande do Sul (COMPESQ) and was conducted following the Law on Procedures for the Scientific Use of Animals - Law No. 11.794 (10/08/2008) and with the norms of the Ethics Committee on the Use of Animals (CEUA) of the Federal University of Rio Grande do Sul.

- Sample Selection Criteria

**Figure 1:**
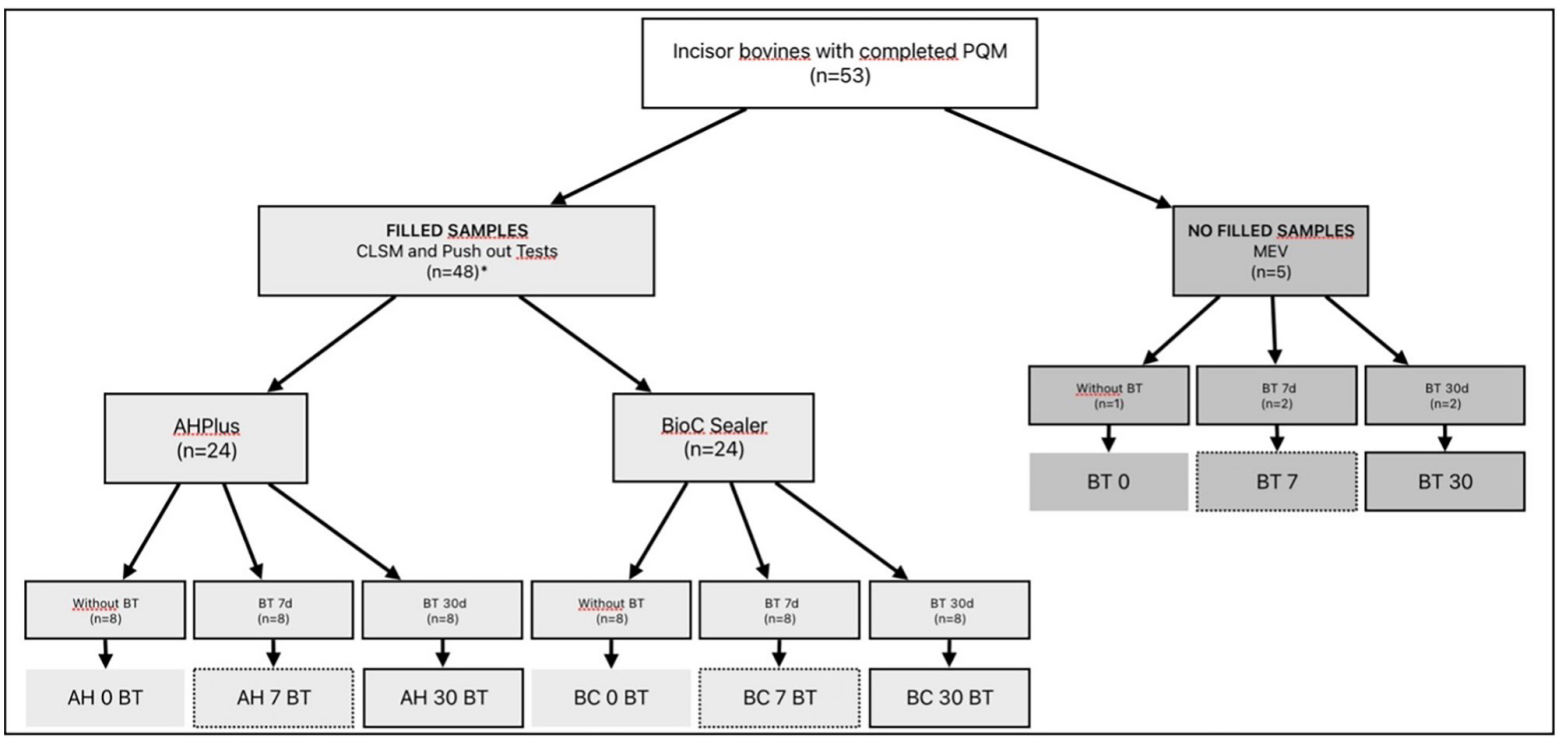
Flowchart with the distribution of filled samples evaluated for intratubular penetration and bond strength and of no filled samples for evaluation of the dentin surface after removal of the BRCD. *The same samples were used both for CLSM and Push out test (Bond Strenght). PQM: biomechanical preparation; n: number of samples; CLSM: Confocal Laser Scanning Microscopy; SEM: Scanning Electron Microscopy; AH0BT: samples filled with AH Plus Sealer without use of Bio C Temp (BT); AH7BT: samples medicated with BT for 7 days and filled with AH; AH30BT: medicated with BT for 30 days and filled with AH; BC0BT: samples filled with Bio-C Sealer (BC) without previous use of BT; BC7BT: samples medicated with BT for 7 days and filled with BC; BC30BT: medicated with BT for 30 days and filled with BC; BT0: sample no medicated with BT and no filled; BT7: samples previously medicated with BT for 7 days and no filled; BT30: samples previously medicated with BT for 30 days and no filled.

Fifty-three bovine incisor teeth were extracted from animals slaughtered for non-research purposes at a slaughterhouse located in Rio Grande do Sul (Brazil). Forty-eight teeth were used for the push-out test and the evaluation of intratubular penetration. The other five teeth were also treated but not filled and evaluated for the presence of intracanal medication remnants by Scanning Electron Microscopy (SEM) (Fig. 1).

After extraction, the teeth were immediately stored in saline solution vials until the use time. The criteria for non-inclusion were: root length less than 15 mm; root fractures or cracks detected through visual examination, more than one root canal, internal or external resorptions verified by previous radiography, obtained with a digital system (Gnatus, São Paulo, Brazil) and incomplete rhizogenesis. All specimens were X-rayed in the vestibule-lingual and mesiodistal directions before preparation to confirm the existence of only one root canal.

- Sample Calculation

The statistical program BioEstat 4.0 (Mamirauá, AM, Brazil) was used to calculate the sample size. It was based on the studies by Kok *et al*. (22) for the analysis of intratubular penetration and Carvalho *et al*. (23) for the analysis of bond strength.

- Experimental procedures

The teeth were sectioned with a diamond disc (FGM, Joiville, Brazil), and the roots were standardized to 15 mm in length. The root canals were explored with a K-15 file (Dentsply-Maillefer, Ballaigues, Switzerland), and the working length was 1 mm below the foraminal opening. This measurement was performed by passively inserting the k-15 instrument into the root canal until it coincided with the foramen. The anatomical diameter of the root canals was determined with a type K instrument passively inserted into the root canal until the instrument was found to be slightly attached to the apical foramen, which was considered the initial apical instrument. The root canals were prepared up to the k#80 file for standardization. During preparation, the root canals were irrigated with a total of 10 ml of 2,5% sodium hypochlorite (NaOCl), with 31-G NAviTipp needles (Ultradent Products Inc, South Jordan, UT) inserted 3 mm short of the apical foramen. The final irrigation was performed using 5 ml of 2.5% NaOCl, followed by 5 ml of saline solution and 5 ml of 17% EDTA (Formula and Action, São Paulo, Brazil) for 3 minutes and again an irrigation with 5 ml of saline solution. The root canals were dried using paper tips corresponding to the file used.

Placement of BRCD: Sixteen teeth were randomly selected for the control group, in which no intracanal medication was used, and these were filled immediately after the chemo-mechanical preparation with two different endodontic sealers. The other thirty-two teeth were selected for the test group, in which BIO-C TEMP (BT) was used as root canal dressing. Syringe tips of BT were positioned at 1 mm of the working length (Fig. 1). A sterile cotton wick was placed at the mouth of the root canal, and it was sealed with Coltosol (Coltène/Whaledent AG Altstätten, Switzerland).

The samples with BT were stored wrapped in moistened gauze in a stove for the sealer to set - 37ºC and 100% humidity for two experimental periods: 7 and 30 days. Sixteen samples remained with BRCD for seven days and sixteen for 30 days (Fig. 1).

- Passive Ultrasonic Activation

The samples were reaccessed after the 7-day and 30-day periods, and the BRCD was removed through passive ultrasonic activation. An Irrisonic ultrasonic tip – E1 20/.01 (Helse Dental Technology, Santa Rosa de Viterbo, São Paulo, Brazil), coupled to an ultrasound unit (Newtron P5 XS SATELEC-ACTEON) with power adjusted according to the manufacturer’s indication. The root canals were filled with saline solution, and the ultrasonic tip was positioned at 2 mm from the working length. Three cycles of activation of 20 seconds (totalizing 1 minute) were performed using 5ml of saline solution, and this substance was changed after each cycle (15ml of saline solution was used in total). At the end of this procedure, the root canals were irrigated with 10 ml of 0.9% saline solution without activation, and then they were dried with absorbent paper points.

- Root canal filling

The 48 teeth were filled, of which 24 were obturated with epoxy resin-based sealer AH Plus (AH) (Ballaigues, Switzerland) and 24 were filled with calcium silicate-based sealer BIO-C Sealer (BC) (Angelus Ind. Londrina, Paraná, Brazil). Figure 1 shows the distribution of the groups according to the sealer used and the time of use of BRCD.

Prior to filling, Rhodamine B (Hexis, Jundiaí, SP, Brazil) was added in the proportion of 0.1% to epoxy resin-based sealer (AP) and 0.1% of Fluo-3 (Thermo Fisher Scientific, USA) to bioceramic sealer (BC) to promote fluorescence and enable CLSM analysis. An analytical balance (Shimadzu, Tokyo, Japan) was used to weigh this. The root canal filling technique used was Lateral Condensation Technique, with the master gutta-percha cones (MK Life, Porto Alegre, Brazil) corresponding to the file used (#80) and accessory gutta-cones (MK Life, Porto Alegre, Brazil), cut and condensed vertically with the FastPack electric condenser (MKlife, Porto Alegre, Rio Grande do Sul, Brazil). The sealers were prepared according to the manufacturer’s recommendation. After root canal filling, the samples were stored in an oven at 37°C and 100% humidity for 14 days.

- Push-Out Test (Bond Strenght Analysis)

After this period, 2 mm thick cuts were made from the apical, medium, and cervical thirds of the root using a cutting machine (Extec Labcut 1010, Enfield, CT). The slices obtained were polished with sanding strips of decreasing grain up to 1200 grit and felt discs with a specific paste (Arotec, Cotia, SP, Brazil). Finally, the samples were washed with distilled water to remove debris from polishing.

To analyze the bonding strength, the slices corresponding to the cervical, middle, and apical thirds were positioned in a metallic device with a central opening (Ø = 3 mm) larger than the diameter of the root canal. The coronary portion of the slice was positioned in contact with the metallic device. Thus, the metal cylinder (Ø = 0.35 mm) induced a load in the down-crown direction on the filling. The push-out test was performed on a universal testing machine (EMIC, São José dos Pinhais, Brazil) at a 1.0 mm/min speed. To obtain the bond strength values, in MPa, the following formula was used: σ = F/A, where F = load required for specimen rupture (N), and A = adhesive area (mm2). To determine the adhesive interface area, a formula to calculate the lateral area of a circular cone with parallel bases was used. The formula was: A=2πg(R1 + R2), where π = 3.14, g = generatrix, R1 = smallest radius of the base, R2 = greater radius of the base. To determine the generatrix, the following calculation was made: g2 = (h2 + [R2- R1]2), where h = height of the sectioned area, R1 and R2 were obtained by measuring the smallest and largest diameters of the base, respectively, which correspond, in turn, to the internal diameter between the root walls of the conduit. These values were obtained using a digital calliper. All data were recorded in a spreadsheet organized for this purpose.

- Confocal Laser Scanning Microscopy (CLSM) for analysis of intratubular penetration

The slices corresponding to the apical third were analyzed using the Olympus Fluoview 1000 confocal laser electron microscope (Olympus Corporation, Tokyo, Japan) with

×10 magnification. The conFigurations used were 70-μm depth and a resolution of 800 x 800 pixels. Low, medium or high intratubular penetration was the criteria to evaluate the extension of the sealer into the dentine.

- Sample Preparation for Scanning Electron Microscopy (SEM)

Five samples were prepared according to the protocol described for bond strength analysis, but they were not filled. One of the samples was used as a control (BT0 group), where BT was not used, and four samples received this medication for 7 (n=2) and 30 (n=2) days (BT7 and BT30 groups, respectively), staying in a greenhouse environment, with 100% humidity at 37 ºC (Fig. 1).

After these periods, the samples were sectioned as follows: first, graphite marking was performed on the buccal and palatal surface of the roots, in the longitudinal direction of the tooth, from cervical to apical, where grooves were made with a double-sided diamond disc at high rotation, without penetrating the canal. The roots were separated by applying pressure with a sealer trowel in the centre of the furrow so that there was no contact between the disc and the part to be analyzed.

- SEM analysis

All samples prepared for the SEM were subjected to a desiccator and then metallized with carbon. One of the halves of each of the samples, chosen randomly, had its dentin microstructure evaluated in the cervical, middle and apical thirds at randomly chosen points, with a magnification of 2.00 K and 10.00 K in a scanning electron microscope - Zeiss EVO-50 (Carl Zeiss AG, Germany) with an acceleration voltage of 10 kV, image resolution of up to 3072 x 2304 pixel. A descriptive analysis of the dentin wall of the root canal was performed based on the presence or absence of remnants of the root canal dressing.

- Statistical Analysis

The Kolmogorov-Smirnov was used to test whether two samples came from the same distribution. The bond strength data were evaluated using non-parametric analysis (Kruskal-Wallis and Dunn’s test) for multiple comparisons and a T-test for comparisons of sealers within the same experimental time. CLSM performed visual descriptive analysis for sealer penetrability, and SEM analyzed the dentin surface of the root canal.

## Results

- Bonding Strength

The bioceramic root canal dressing BioC Temp decreased the bond strength of both sealers tested (AH and BC) in both experimental times (7 and 30 days) (*P*< 0.05). When the root third was considered, only in the middle third occurred a significant decrease in the bond strength between AH0BT and AH7BT and between BC 0 BT and BC 30 BT (*P*< 0.05). There were no significant differences in the intra-thirds root analysis (*P* > 0.05). The results of the push-out test are described in  Table 1.

- Intratubular penetration by CLSM

**Figure 2:**
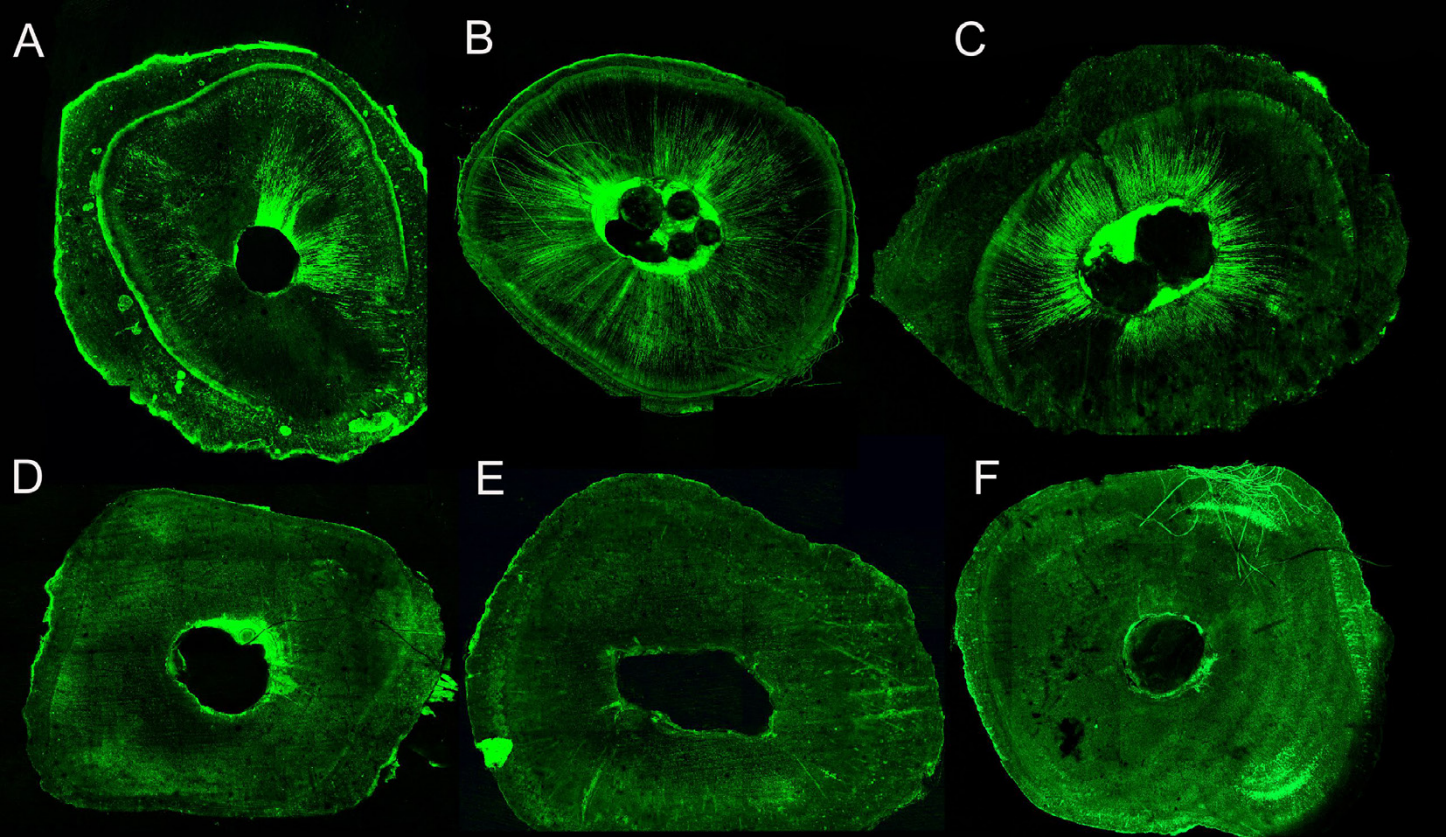
Penetration of endodontic sealers in CLSM. A: AH0BT: samples filled with AH Plus Sealer without use of Bio C Temp (BT); B: AH7BT: samples medicated with BT for 7 days and filled with AH; C: AH30BT: medicated with BT for 30 days and filled with AH; D: BC0BT: samples filled with Bio-C Sealer (BC) without previous use of BT; E: BC7BT: samples medicated with BT for 7 days and filled with BC; E: BC30BT: medicated with BT for 30 days and filled with BC.

The analysis of the CLSM images showed that the intratubular penetration of AH was higher than the BC, according to Figure 2.

- SEM analysis

**Figure 3:**
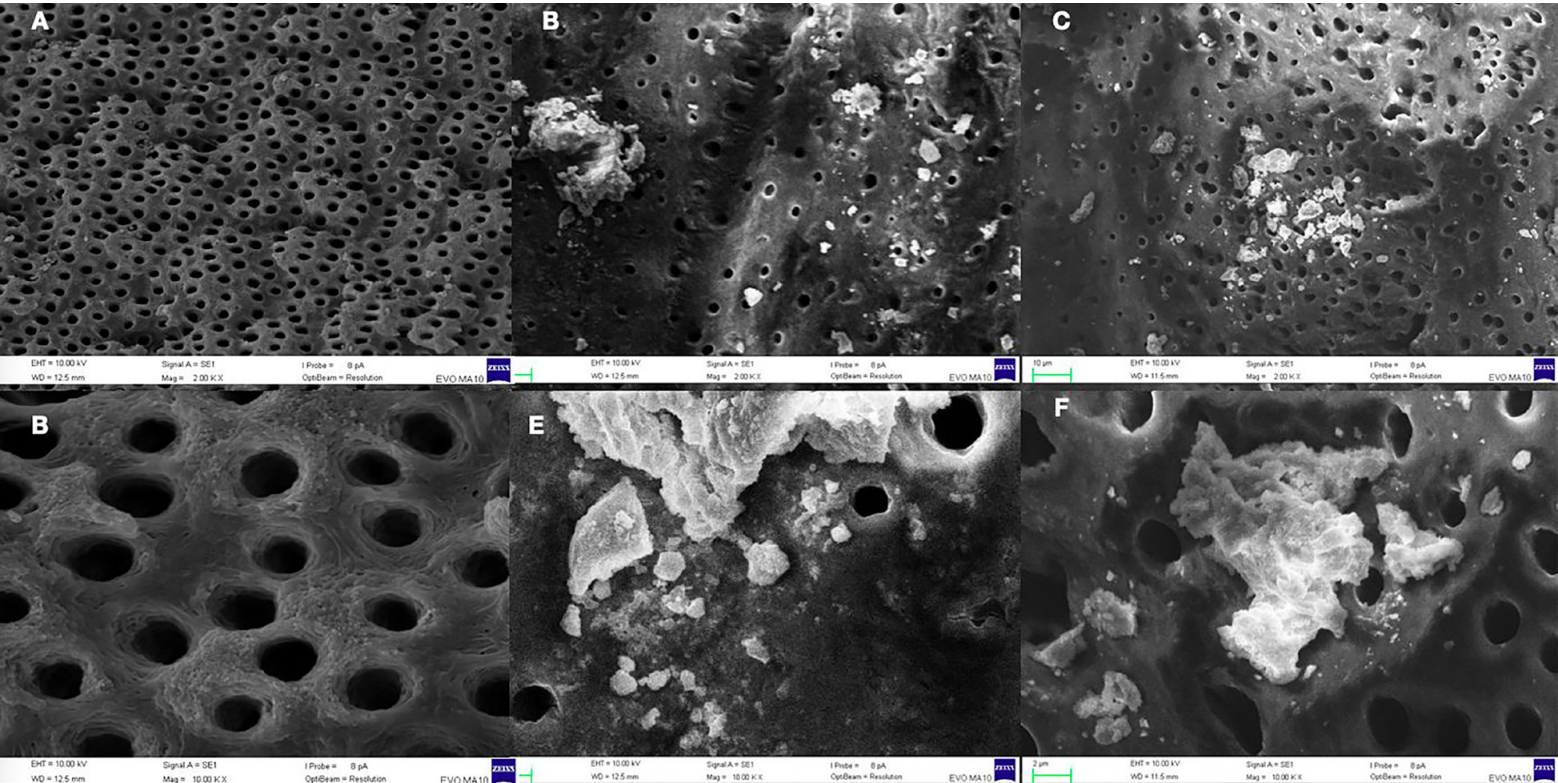
Electromicrographs of the dentin surface without BT and after removal of BT at 7 and 30 days. A and D: dentin surface of BT0 group (without use of BRCD), at 2.00K (A) and 10.00 K (D) magnification, showing dentinal tubules open, free of debris or medication residues. B and E: dentin surface of BT7 group (medicated with BT for 7 days and after ultrasonic activation with saline solution), at 2K magnification and 10K magnification. C and F: dentin surface of BT30 group (medicated with BT for 30 days and after passive ultrasonic activation with saline solution), at 2K and 10K magnification. It is possible to see medication residues in both groups, BT7 (7 days of BRCD use) and BT30 (30 days of BRCD use).

In this analysis, despite using ultrasound, BRCD was observed on the dentin walls in both test groups (7 and 30 days) (Fig. 3).

## Discussion

In the present study, the bond strength, the intratubular penetrability of two types of endodontic sealers and the analysis of the root canal dentin surface by SEM were evaluated when BT was used as root canal dressing. The control group was defined as the group in which no intracanal medication (IM) was used so that the dentin without residue could serve as a comparison parameter in order to evaluate possible bioactivity and presence of medication remnants that could interfere with the bond strength and penetrability of the sealers when using BT.

According to the manufacturer, the material contains calcium silicate particles, which hydrate in water, forming hydrated calcium silicate on its surface. However, the polymeric chains present in the product prevent the connection with the hydrated particles, thus restricting the hardening of the material, which would facilitate removal since this material is for temporary use and needs to be removed from the interior of the root canal. However, it is known that it is impossible to remove the medication from the inside of the root canal (4), even if ultrasonic activation is used. This fact was observed in the SEM analysis, which was present independently of the root third.

Based on the findings, the null hypothesis was rejected since the BRCD interfered with the bond strength of the endodontic sealers to the dentin. This was probably due to the remnants of intracanal medication in the dentin walls, which obliterated the tubules and thus decreased the bond strength.

The push-out test, used to evaluate bond strength, has been considered an appropriate method for evaluating the bond strength of filling materials to root dentin (24-26). The results of this study showed that the use of BRCD decreased the bond strength of both AH Plus and Bio-C Sealers. This result is not in line with a recent study (27) that showed that intracanal medication based on calcium silicate could chemically interact with bioceramic sealer, forming a biomineralizing layer increasing bond strength; this was not observed in this experiment: the lowest bond strength values were observed for teeth that received BT, regardless of the filling sealer.

Sealer penetration into dentin tubules can prevent the proliferation of residual bacteria and root canal recontamination (28). Two markers, Rhodamine B and Fluo-3, were used as fluorophores.

Rhodamine B emits red fluorescence and was used in the AH-filled group. It has a high affinity for moisture and a lower affinity for calcium (29). Due to its high affinity for humidity, if used with a bioceramic sealer, which is a hydraulic sealer, it would cause a confounding factor since it would not be possible to assess penetrability accurately, as the marker could evidence the water present and generate a false-positive result (30).

Fluo-3 is a selective fluorescent marker for calcium ions (31), which has been indicated to promote fluorescence in silicate and calcium oxide-based materials (32). Thus, the calcium present in the intracanal medication binds to Fluo-3, and its fluorescence, in a shade of green, observed in the confocal microscopy images, increases according to the stability of the bonds formed, evidencing the real penetrability of the calcium silicate-based sealer.

Despite remnants of the bioceramic medication, the AH Plus sealer showed more evident intratubular penetration than the Bio-C sealer. Possible explanations may be related to the flow, viscosity, solubility, and particle size of the sealers tested (33), organic and inorganic content in the dentin tubule, and greater anatomical complexity of the apical third (34). Our results agree with Lemos *et al*. (35), which showed slight intratubular penetration of a bioceramic sealer with Fluo 3 as a marker. On the contrary, El Hachen *et al*. (36) found higher intratubular penetration values for bioceramic sealers compared to AH. However, these authors used Rhodamine B as fluorophore for both sealers, which could be a false positive result.

In addition, a remnant of BT was observed in the root canal dentin walls in both groups analyzed by SEM. These remnants formed a physical barrier that may have hindered the sealers’ adhesion to the dentin, reducing the bond strength and justifying the findings of the push-out test (7,37).

The main limitation of our study is the impossibility of transporting our laboratory results to the clinic. However, they can be a starting point for a better understanding of a bioceramic material used as root canal dressing. Likewise, we do not intend to indicate such material as a substitute for calcium hydroxide pastes since they have already overcome the barrier of time with strong scientific evidence.

## Conclusions

Therefore, based on the results of this study, it is possible to conclude that the AH Plus sealer showed more evident intratubular penetration than the Bio-C sealer. It is not possible to altogether remove BRCD from the canal. A bioceramic root canal dressing, Bio-C Temp, decreased the bond strength of both tested sealers: epoxy resin AH Plus and bioceramic Bio-C Sealer.

## Figures and Tables

**Table 1:** Bond strength (MPa) of AH Plus (AH) and Bio-C Sealer (BC) to the dentin walls, without BRCD Bio-C Temp (BT) and after 7 and 30 days BT use.

Groups	Median and 25th and 75th percentiles			
	Root canal third			Total
	Cervical	Medium	Apical	
AH0BT	1.36^Aa^ (1.16-1.78)	3.44^Aa^(1.49-4.17)	3.46^Aa^(2.13-5.23)	2.62^A^ (1.84-3.51)
AH7BT	0.97^Aa^^(^0,73-1,16)	1.09^Ba^(0.63-1.37)	1.88^Aa^(1.32-2.08)	1.27^B^(1.01-1.57)
AH30BT	0.89^Aa^(0.56-1.12)	1.34^ABa^(0.92-1.65)	1.88^Aa^(1.69-1.98)	1.42^B^ (1.07-1.82)
BC0BT	2.76^Aa^(1.02-3.82)	3.37^Aa^(1.26-4.34)	3.86^Aa^(2.01-5.30)	2.95^A^(1.97-4.54)
BC7BT	0.89^Aa^(0.49-1.15)	0.96^Aa^(0.13-1.64)	1.84^Aa^(0.36-3.67)	0.68^B^(0.51-2.30)
BC30BT	0.57^Aa^(0.36-1.29)	0.49^Ba^(0.38-0.75)	1.10^Aa^(0.50-3.56)	0.93^B^(0.60-1.44)

Equal capital letters on the column do not differ statistically by the Kruskal-Wallis test. Equal lowercase letters in the line do not differ statistically by the Kruskal Wallis test. *P*< 0.05.

## Data Availability

The datasets used and/or analyzed during the current study are available from the corresponding author.
